# Characterization of Four Bifunctional Plant IAM/PAM-Amidohydrolases Capable of Contributing to Auxin Biosynthesis 

**DOI:** 10.3390/plants3030324

**Published:** 2014-08-07

**Authors:** Beatriz Sánchez-Parra, Henning Frerigmann, Marta-Marina Pérez Alonso, Víctor Carrasco Loba, Ricarda Jost, Mathias Hentrich, Stephan Pollmann

**Affiliations:** 1 Center for Plant Biotechnology and Genomics (U.P.M.-I.N.I.A.), Technical University Madrid, Montegancedo Campus, Crta. M-40, km 38, 28223 Pozuelo de Alarcón (Madrid), Spain; E-Mails: beatriz.sanchez@upm.es (B.S.-P.); martamarina.perez@upm.es (M.-M.P.A.); victorcarrascoloba@gmail.com (V.C.L.); 2 Department of Plant Physiology, Faculty of Biology and Biotechnology, Ruhr-University Bochum, Universitätsstraße 150, 44801 Bochum, Germany; E-Mails: henning.frerigmann@uni-koeln.de (H.F.); mathias.hentrich@rub.de (M.H.); 3 School of Plant Biology, University of Western Australia, 35 Stirling Highway, Crawley, WA 6009, Australia; E-Mail: ricarda.jost@uwa.edu.au

**Keywords:** amidase, auxin, indole-3-acetic acid, indole-3-acetamide, phenyl-2-acetic acid, phenyl-2-acetamide

## Abstract

Amidases [EC 3.5.1.4] capable of converting indole-3-acetamide (IAM) into the major plant growth hormone indole-3-acetic acid (IAA) are assumed to be involved in auxin *de novo* biosynthesis. With the emerging amount of genomics data, it was possible to identify over forty proteins with substantial homology to the already characterized amidases from *Arabidopsis* and tobacco. The observed high conservation of amidase-like proteins throughout the plant kingdom may suggest an important role of theses enzymes in plant development. Here, we report cloning and functional analysis of four, thus far, uncharacterized plant amidases from *Oryza sativa*, *Sorghum bicolor*, *Medicago truncatula*, and *Populus trichocarpa*. Intriguingly, we were able to demonstrate that the examined amidases are also capable of converting phenyl-2-acetamide (PAM) into phenyl-2-acetic acid (PAA), an auxin endogenous to several plant species including *Arabidopsis*. Furthermore, we compared the subcellular localization of the enzymes to that of *Arabidopsis* AMI1, providing further evidence for similar enzymatic functions. Our results point to the presence of a presumably conserved pathway of auxin biosynthesis via IAM, as amidases, both of monocot, and dicot origins, were analyzed.

## 1. Introduction

The collective term auxin refers to a class of compounds sharing similar physiological functions in plants. Auxins are well characterized as growth promoting phytohormones, occurring at low concentrations across plant species. They are known to be essential for the regulation of a wealth of different processes, such as cell elongation, initiation of lateral and adventitious root growth, flower and fruit development, and fruit ripening [1,2]. Albeit indole-3-acetic acid (IAA) is considered the major auxin in plants, a number of additional naturally occurring compounds that exert auxin effects have been reported to date (e.g., 4-chloroindole-3-acetic acid [3], and phenyl-2-acetic acid (PAA) [4,5]). Moreover, a variety of IAA precursors such as indole-3-acetamide (IAM), indole-3-acetonitrile, indole-3-pyruvic acid (IPyA) [1,6], and indole-3-butyric acid [7,8] exhibit auxin-like effects, most likely due to their conversion to IAA.

**Figure 1:**
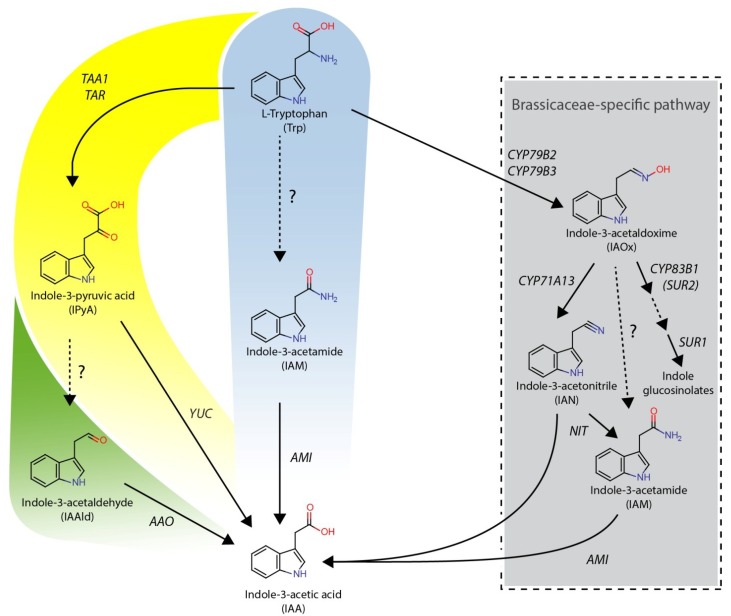
Proposed pathways of L-tryptophan-dependent IAA biosynthesis in plants. The IAOx-pathway that is seemingly restricted to indole glucosinolate-producing plant species is given in the grey box. In yellow, the IPyA-pathway is shown; a possible side-branch (IAAld-pathway) is added in green to the IPyA-pathway. In the middle, the IAM-pathway is highlighted in blue. Dashed lines indicate assumed reaction steps for which the corresponding enzymes have yet to be identified.

Although much is known about the physiological functions and effects of auxins, the biosynthesis of substances of this compound class still remains partially elusive (Figure 1).

The main portion of free IAA in plants is seemingly produced via a two-step pathway involving tryptophan aminotransferases (TAA1 and TARs) and flavin-containing monooxygenases of the YUCCA family (YUC1-11) [9,10]. However, there is additional evidence that the biosynthesis of IAA, at least to a certain extent, may also proceed via a number of other metabolic routes, which are classified in terms of their intermediates. These routes are thought to act either in parallel or in a developmentally regulated manner [11,12,13,14]. One of these biosynthetic pathways is supposed to lead from L-tryptophan via IAM to IAA and appears to be one of the two major pathways commonly used in bacteria to produce IAA [15,16,17]. Previous work provided growing evidence for the existence of a comparable IAM-pathway to be operative *in planta* as well, suggested by the following observations: (i) in a number of independent studies IAM has been identified as a natural constituent of several plant species including *Citrus unshiu* [18], *Prunus jamasakura* [19], *Curcubita maxima* [20], as well as in *Arabidopsis thaliana*, *Oryza sativa*, *Zea mays*, and *Nicotiana tabacum* [21,22]; (ii) IAM hydrolyzing activities have been reported from *Triticum* *aestivum* and *Pisum sativum* (whole plant extracts) [6], *O.* *sativa* (callus extracts) [23,24], as well as from *Poncirus trifoliate* (fruit extracts) [25]; and (iii) amidases from *A. thaliana* and *N. tabacum* capable of converting IAM to IAA* in vitro* have been identified [26,27].

Enzymes are abbreviated as follows: AAO, ARABIDOPSIS ALDEHYDE OXIDASE 1; AMI, AMIDASE; CYP71A13, CYTOCHROME P450 MONOOXYGENASE 71A13; CYP79B2/B3, CYTOCHROME P450 MONOOXYGENASE 79B2/B3; CYP83B1, CYTOCHROME P450 MONOOXYGENASE 83B1; NIT, NITRILASE; SUR1, SUPERROOT 1 (S-ALKYL-THIOHYDROXIMATE LYASE); TAA1, TRYPTOPHAN AMINOTRANSFERASE OF ARABIDOPSIS 1; TAR2, TRYPTOPHAN AMINOTRANSFERASE RELATED 2; YUC, YUCCA.

Like the bacterial IAM-hydrolases from *Agrobacterium tumefaciens* [28] and *Pseudomonas syringae* [29], *At*AMI1 is a member of the amidase-signature superfamily, characterized by a glycine and serine rich stretch of approximately 50 amino acids, which contains most of the catalytically relevant amino acid residues [30]. *At*AMI1 is located in both the cytoplasm and nucleoplasm, acts as an obligate monomer of approximately 46 kDa, and is mainly expressed in tissues with high meristematic activity, for instance young leaves and floral buds [31,32]. Moreover, *At*AMI1 specifically converts IAM to IAA* in vitro*, while refusing most other naturally occurring amides as substrates. On this basis, a tentative involvement of *At*AMI1 in auxin biosynthesis in *A. thaliana* has been suggested.

Due to these lines of evidence, implying a possibly broader importance of IAA biosynthesis through the IAM-pathway in plants, it appeared intriguing to us to investigate whether IAM-amidohydrolases are restricted to only very few plant families or if this enzyme class shows a broader distribution in the plant kingdom. The identification and functional analysis of comparable AMI1-like enzymes from various plant species would likewise highlight a broader presence of the IAM-dependent IAA production and point towards a general concept in auxin biosynthesis.

Thoroughly analyzing the wealth of emerging genome data from plants, we were able to identify a reasonably high number of AMI1-like proteins. Phylogenetic comparison revealed substantial sequence homology between those proteins and *Arabidopsis* AMI1. To gain further insight into the function of these enzymes, we chose four amidases from *O. sativa*, *Sorghum bicolor*, *Medicago truncatula*, and *Populus trichocarpa* in this study and compared them to *At*AMI1 [26,31]. With respect to enzymatic properties and subcellular localization, we observed considerable similarity between the four candidate enzymes and *At*AMI1, although the selected amidases displayed lower specific activities towards IAM when compared to the properties of *At*AMI1. Furthermore, we were capable of demonstrating that all examined amidases are additionally able to convert phenyl-2-acetamide (PAM) to the naturally occurring auxin, PAA. We also show that PAA is endogenous to *Arabidopsis*, and that it exerts an auxin-like effect in *Arabidopsis* root growth bioassays.

## 2. Results and Discussion

### 2.1. Identification of AMI1-like Proteins in Plant Genomes

**Figure 2:**
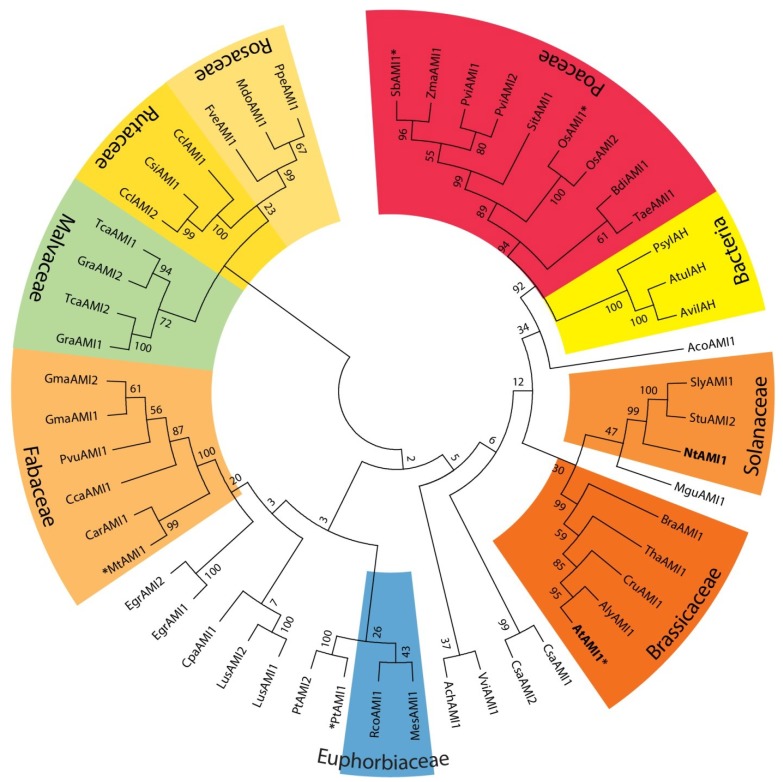
Cladogram of 51 AMI1-like proteins from plants and bacteria.

Homologs of the *Arabidopsis* *YUC* gene family have been identified and characterized from several plant species including petunia, rice, tomato, maize, and pea [33,34,35,36,37], suggesting a widespread occurrence of the YUC-dependent IAA biosynthetic pathway in the plant kingdom. In contrast, CYP79B2/B3 and NIT1-3 enzymes seem to be restricted to the *Brassicaceae* [22,38,39], which contradicts a general importance of these enzymes in auxin biosynthesis. To investigate the distribution of AMI1-like proteins in the plant kingdom, the *Arabidopsis* IAM-hydrolase (*At*AMI1, At1g08980) nucleic acid sequence, as well as the translated primary amino acid sequence, were used to query publicly available databases (e.g., Phytozome, Plant Gene Duplication Database) for candidate IAM-hydrolase orthologs. The *in silico* analyses provided indication for 47 *At*AMI1-like proteins from 38 plant species (Figure 2).

It is noteworthy to mention that, at least to the best of our current knowledge, all plant genomes published thus far contain one or more *At*AMI1-like sequences, which may suggest an important function of this enzyme class in plant development, as the gene is conserved across a large number of plant species. The notion of a wider distribution of AMI1-like proteins in higher plants is further strengthened by the previously mentioned identification of IAM and detection of IAM-hydrolase activities in various other plant species, respectively. In the first place, however, the obtained results indicate a broader distribution of *At*AMI1-like proteins in the plant kingdom.

**Figure A1:**
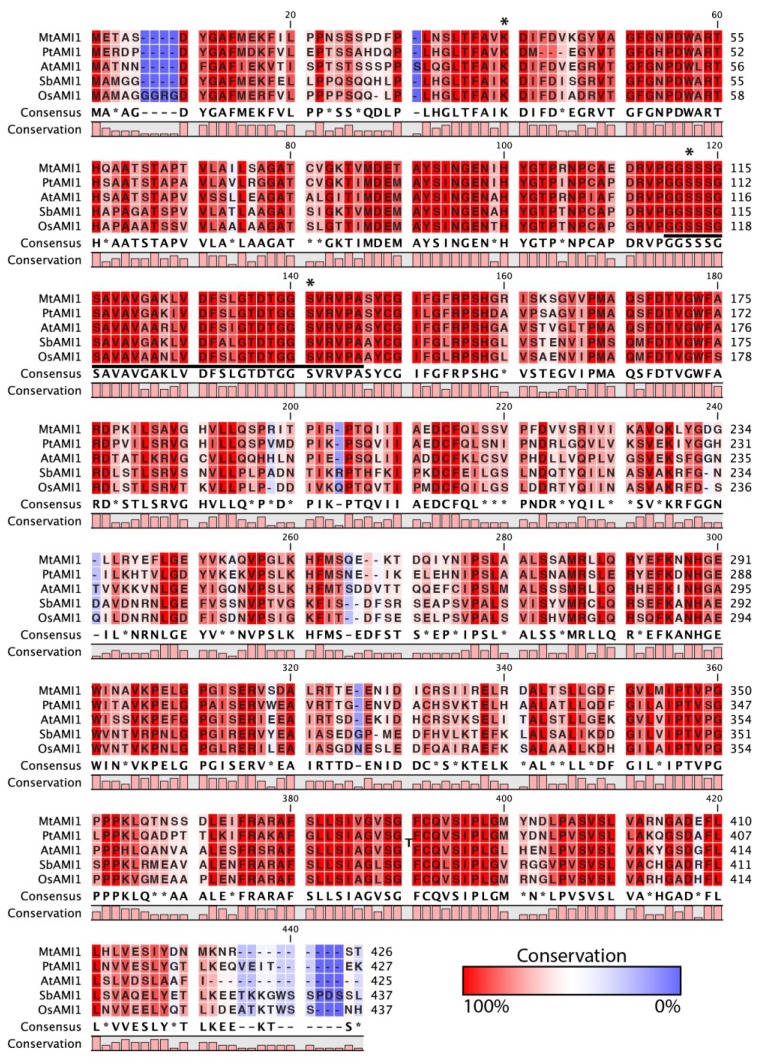
Sequence alignment of five members of the amidase-signature family from plants. The analyzed sequences are *At*AMI1 (At1g08980), *Mt*AMI1 (Mt1g099370), *Os*AMI1 (Os04g02780), *Pt*AMI1 (Pt13g02200), and *Sb*AMI1 (Sb02g039510). Amino acid positions are indicated. Sequence conservation among the polypeptides is color-coded in the alignment. Asterisks mark the catalytic triad; the glycine- and serine-rich amidase-signature motif is underlined.

A bootstrap consensus tree inferred from 500 replicates was generated to represent the phylogenetic relationships of the proteins analyzed. Proteins from the plant species highlighted in bold letters have already been enzymatically characterized in our group [26,30,31] or by other laboratories [27]. The amidases further studied in this work are labeled with asterisks. An amino acid sequence alignment of the selected enzymes can be taken from Appendix Figure A1. Plant species that belong to the same family are given in colored boxes. Species names are abbreviated as follows with gene/protein identifiers given in parenthesis: Ach: *Actinidia chinensis* (ACHN083681); Aco: *Aquilegia coerulea* (Aquca_05400135.1); Aly: *Arabidopsis lyrata* (16043473); At: *Arabidopsis thaliana* (At1g08980); Atu: *Agrobacterium tumefaciens* (YP_001967410); Avi: *Agrobacterium vitis* (P25016); Bdi: *Brachypodium distachyon* (Bradi5g27490); Bra: *Brassica rapa* (Bra031652); Car: *Cicer* *arietinum* (XP_004495736); Cca: *Cajanus* *cajan* (C.cajan06830); Ccl: *Citrus clementine* (AMI1: Ciclev10001239m, AMI2: Ciclev10001213m); Cpa: *Carica papaya* (evm.model.supercontig_48.34); Cru: *Capsella rubella* (Crubv10006860m); Csa: *Cucumis sativus* (AMI1: Cucsa.11590.1, AMI2: Cucsa.182140.1); Csi: *Citrus sinensis* (orange1.1g044294m); Egr: *Eucalyptus grandis* (AMI1: Eucgr.B01994.1, AMI2: Eucgr.B01996.1); Fve: *Fragaria vesca* (Mrna22572.1-v1.0-hybrid); Gma: *Glycine max* (AMI1: Glyma10g29640.1, AMI2: Glyma20g37660.1); Gra: *Gossypium raimondii* (AMI1: Gorai.007G295700.1, AMI2: Gorai.007G295900.1); Lus: *Linum usitatissimum* (AMI1: Lus10029941, AMI2: Lus10004466); Mdo: *Malus x domestica* (MDP0000302761); Mes: *Manihot esculentum* (cassava4.1_010118m); Mgu: *Mimulus guttatus* (mgv1a006860m); Mt: *Medicago truncatula* (Medtr1g082750.1); Nt: *Nicotiana tabacum* (AB457638); Os: *Oryza sativa* (AMI1: Os04g02780.1, AMI2: Os04g02754.1); Psy: *Pseudomonas syringae* (P52831); Ppe: *Prunus persica* (ppa005982m); Pt: *Populus trichocarpa* (AMI1: Potri.013g024100.1, AMI2: Potri.013g024200.1); Pvi: *Panicum virgatum* (AMI1: Pavirv00043873m, AMI2: Pavirv00038995m); Pvu: *Phaseolus vulgaris* (Phvul.007G180900.1); Rco: *Ricinus communis* (30128.m008759); Sb: *Sorghum bicolor* (Sb02g039510.1); Sit: *Setaria italica* (Si001473m); Sly: *Solanum lycopersicum* (Solyc10g086170.1.1); Stu: *Solanum tuberosum* (PGSC0003DMP400065555); Tae: *Triticum aestivum* (AK330138); Tca: *Theobroma cacao* (AMI1: Thecc1EG046817t1, AMI2: Thecc1EG046818t1); Tha: *Thellungiella halophile* (Thhalv10007679m); Vvi: *Vitis vinifera* (GSVIVT01000036001); Zma: *Zea mays* (GRMZM2G169087_T01).

### 2.2. Subcellular Localization of Selected Plant Amidases

The high degree of sequence homology between the *At*AMI1-like proteins compared in Figure 2 made it tempting to speculate that the identified enzymes also share similar functions. In order to gain deeper insight into their functional properties, we selected four candidate amidases both from monocot and dicot species, namely *O. sativa* *Os*AMI1 (Os04g02780), *S. bicolor* *Sb*AMI1 (Sb02g039510), *M. truncatula* *Mt*AMI1 (Mt1g099370), and *P. trichocarpa* *Pt*AMI1 (Pt13g02200), for further investigation. On a protein level, the selected candidates show an overall amino acid identity of 57% (*Os*AMI1), 58% (*Sb*AMI1), 66% (*Mt*AMI1), and 68% (*Pt*AMI1), respectively, relative to *At*AMI1. As shown in Appendix Figure A1, in particular the region of the amidase signature, containing most of the catalytically relevant amino acid residues [30], shows a high degree of amino acid identity. Consequently, there is reason to suspect that they share similar function.

**Figure 3:**
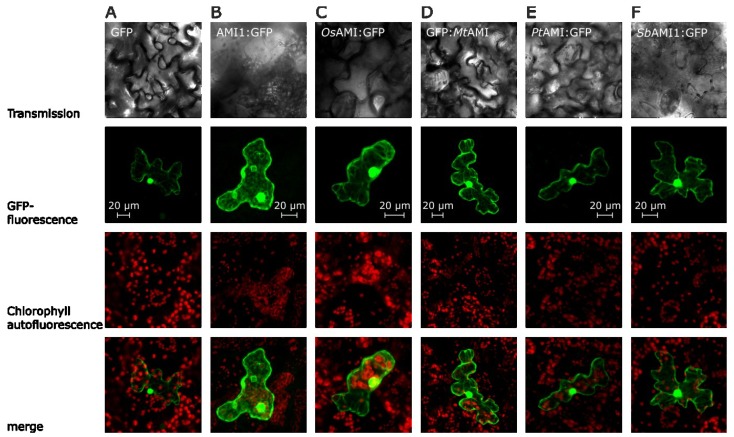
Localization of amidase GFP fusion proteins in epidermal cells of *Arabidopsis thaliana*. The images show typical results of confocal laser scanning microscopic studies, using differential emission light filter sets. The upper row provides transmission images, followed by the GFP channel (GFP-fluorescence), showing the fluorescence of the GFP-fluorophore between 500 and 530 nm. Next, the chlorophyll-autofluorescence is shown in a separate channel (650 to 798 nm). In the bottom row, an overlay of the fluorescence channels is depicted. (**A**) Transformation with an empty GFP-vector (pSP-EGFP), cytoplasmic control; (**B**) Transformation with an *AMI1*:*GFP* construct from *A. thaliana* [31] (positive control); (**C**) Transformation of an epidermis cell with the *Os*AMI1:GFP construct; (**D**) Transformation with the chimeric GFP:*Mt*AMI construct; (**E**) Transformation with the *Pt*AMI:GFP vector; (**F**) Transformation of the *Sb*AMI1:GFP construct. Scale bars for each set of pictures are included in the figure.

In a first set of experiments, the subcellular localization of chimeric GFP fusion proteins was investigated in transient assays. In each experiment, 5 μg of plasmid DNA, facilitating the *35S*-promoter driven expression of the corresponding fusion construct, were transferred into leaf epidermal cells of *A. thaliana* by particle bombardment. Like the AMI1 enzyme from *A. thaliana*, the amidases from *O. sativa*, *S. bicolor*, *M. truncatula*, and *P. trichocarpa*, appear located to the cytoplasm of epidermal cells when transiently expressed as GFP fusion constructs. Moreover, the microscopic assessment of the intracellular protein localization shown in Figure 3 provided further evidence for a diffusion of all studied amidase-constructs into the nucleus and an accumulation in the nuclear pocket, thus, delivering results similar to those obtained for the* Arabidopsis* AMI1:GFP fusion construct that has been shown to be able to diffuse through nuclear pores. However, as previously shown, the nuclear localization of *At*AMI1 is most likely not by reason of a specific nuclear import, but rather due to the compact conical shape of the protein [30,31].

The obtained results are consistent with predictions of the TargetP [40] and PredictNLS server [41], detecting neither nuclear localization signals or signals for the secretory pathway nor mitochondrial and plastidial import signals in the primary amino acid sequence of the analyzed enzymes. In any case, the similar cellular localization pattern of the tested amidases adds an additional cue highlighting a possibly comparable function of the polypeptides.

### 2.3. Functional Analysis of the Selected Plant Amidases

**Figure 4:**
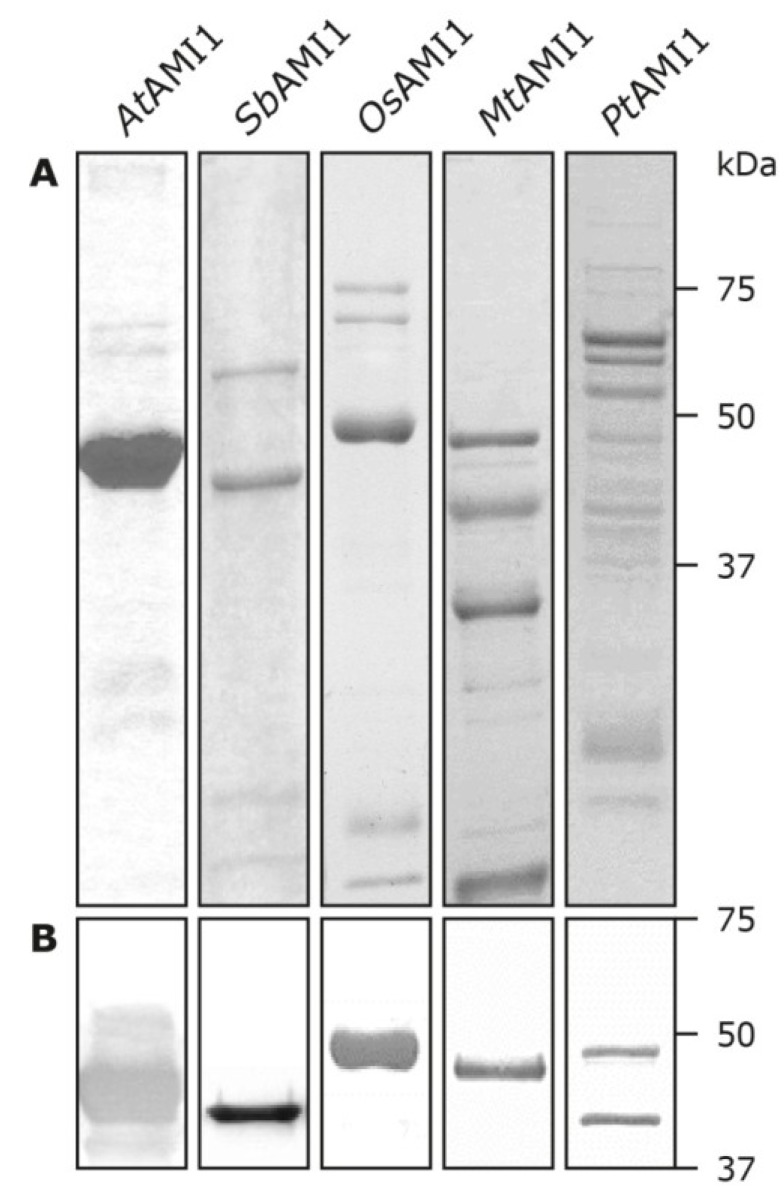
Expression and purification of recombinant amidases. Hexahistidine tagged amidases were expressed in *E. coli* and purified as described in the experimental section. Aliquots of 12.5 μL of the corresponding elution fractions were used for SDS-polyacrylamide gel electrophoresis and subsequent coomassie-staining (**A**) and immunoblotting (**B**). For the immunoblot only the region between 75 and 37 kDa is shown.

To assess whether the proteins possess IAM-amidohydrolase activity* in vitro*, the respective cDNAs from *O. sativa*, *S. bicolor*, *M. truncatula*, and *P. trichocarpa* were amplified by RT-PCR and cloned into the prokaryotic expression vector pTrcHis2 (Invitrogen) for in-frame expression with a c-myc/hexahistidine double tag in *Escherichia coli*, strain BL21-AI (Invitrogen). Recombinant proteins were isolated from bacterial lysates by Ni^2+^-nitrilotriacetate affinity chromatography. As displayed in Figure 4, due to general difficulties in expressing amidases in bacteria, the amount as well as the homogeneity of the purified protein fractions varied considerably. For this reason, the measured enzymatic activities have to be taken with some caution.

**Figure 5:**
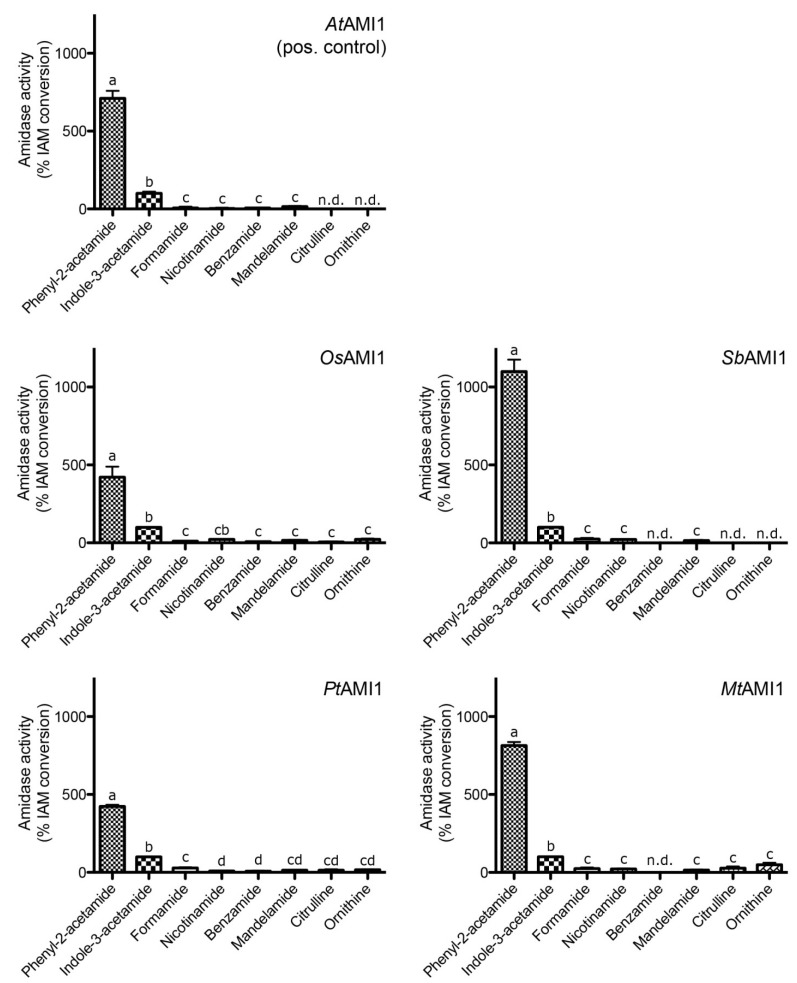
Analysis of the relative substrate conversion of recombinant amidases.

In order to compare the biochemical properties of the recombinant amidases to those of *At*AMI1 in terms of specific activity and substrate selectivity, equal amounts of total protein (5 μg each) from desalted elution fractions were utilized. In this series of experiments, substrate concentrations of 10 mM were used. The data for the substrate specificity are shown on a relative scale normalized to the activity towards IAM (100%) (Figure 5).

Generally, the substrate preferences of the amidases from *O. sativa*, *S. bicolor*, *M. truncatula*, and *P. trichocarpa* were similar, but not identical, to those of *At*AMI1. Under the used conditions, the best substrate found was phenyl-2-acetamide (PAM), a compound that is endogenous to *Phaseolus mungo* [42,43], while an occurrence in *A. thaliana* has not yet been described. *In planta*, PAM might arise from turnover or breakdown of glucotropaeolin, a L-phenylalanine derived benzylglucosinolate [44]. The product of the amidase catalyzed PAM conversion, phenyl-2-acetic acid (PAA), has been shown to occur in a number of different plant species, including *P. mungo* [45], *Solanum lycopersicum*, *N. tabacum*, and *Z. mays* [4], as well as *Tropaeolum majus* [5], but to our knowledge, occurrence of PAA in *A. thaliana* has not yet been reported.

All assays were performed in a total reaction volume of 300 μL at pH 7.5 and 30 °C (substrate concentration 10 mM, reaction time 4 to 6 h). All activities were normalized to the conversion of IAM (100%). Catalytic activities are corrected for background activities obtained for the non-enzymatic breakdown of substrates in heat-inactivated and empty-vector control samples. Maximum specific activities for IAM were 3070 pkat (mg protein)^−^^1^ (*At*AMI1), 2378 pkat (mg protein)^−^^1^ (*Sb*AMI1), 375 pkat (mg protein)^−^^1^ (*Os*AMI1), 37 pkat (mg protein)^−^^1^ (*Mt*AMI1), and 329 pkat (mg protein)^−^^1^ (*Pt*AMI1). The data shown are means ± SE derived from *n* ≥ 3 experiments. n.d.: not detected. Mean values within a graph are significantly different (*p* < 0.05) where superscript letters differ.

To further explore the enzymatic parameters of the enzymes, pH, as well as temperature optima, were estimated. These data are given, together with the specific activities, in Table 1. All enzymes displayed highest conversion rates at a pH between 6 and 7.5, characteristic for cytosolic proteins, confirming the previously described intracellular localization of the proteins. Relative to the other compared polypeptides, *Os*AMI1 exhibited a slightly lower temperature optimum, at 27 °C, which might be explained by differences in the plants’ habitat. Whereas *A. thaliana*, *S. bicolor*, *P. trichocarpa* and *M. truncatula* grow on dry land, *O. sativa*, as a helophyte, has a high tolerance towards flooding. For the latter, evaporation of water from the paddy fields, accounting for a slightly cooler microclimate [46], might be the reason for an evolutionary adjustment of the enzymatic parameters to higher activities at lower temperatures. However, when trying to determine the *K*_m_ values for IAM, it was found that none of the enzymes followed a classical Michaelis-Menten kinetics but rather followed a sigmoidal curve progression, which resemble the results obtained for *At*AMI1 [26], and might indicate an allosteric mode of action. Due to solubility constraints of the substrates and inhibitory effects of the organic solvent (either methanol or ethanol), substrate concentrations of more than 25 mM could not be assessed. At this concentration no saturation of the enzymatic reaction could be observed, thus, preventing the calculation of apparent *K*_m_ values (data not shown). In most instances, the analyzed amidases displayed quite similar characteristics to *At*AMI1, showing comparable pH and temperature optima, although some distinct features were found as well. Most strikingly, the amidases from both *O. sativa* (*Os*AMI1) and *P. trichocarpa* (*Pt*AMI1) showed an approximately ten-fold decrease in specific activity towards IAM, whereas the amidase of *M. truncatula* (*Mt*AMI1) displayed a 100-fold decrease in specific activity for the same substrate* in vitro*. Nevertheless, the examined polypeptides showed clear amidase activity, converting preferentially PAM and IAM relative to other tested amides. In assays with empty vector controls and heat-denatured protein fractions, respectively, no considerable substrate conversion was detected. The highest background activity for the non-enzymatic conversion of the amide substrates to the corresponding free acids was detected for formamide and benzamide, with 0.14% and 0.11% of the enzymatic IAM conversion, respectively. A summarized overview of the enzymatic parameters is given in Table 1.

**Table 1:** Comparison of the enzymatic parameters obtained for recombinant amidases from *S. bicolor*, *O. sativa*, *P. trichocarpa*, and *M. truncatula* with those of the *A. thaliana* AMI1 ^a^.

Parameter	*At*AMI1	*Sb*AMI1	*Os*AMI1	*Pt*AMI1	*Mt*AMI1
Amino acid identity to *At*AMI1 [%]	100	58	57	64	66
Intracellular localization	cytoplasm/nucleoplasm	cytoplasm/nucleoplasm	cytoplasm/nucleoplasm	cytoplasm/nucleoplasm	cytoplasm/nucleoplasm
Specific activity (IAM) [pkat mg^−1^]	3070 ± 520	2378 ± 324	375 ± 52	329 ± 22	37 ± 3
Temperature optimum [°C]	37	45	27	37	35
pH optimum	7.5	6	7.5	7.5	7.5
Calculated molecular weight [kDa]	46	45	50	53	57

^a^ The data shown are means ± SE from at least three independent experiments.

In summary, besides a cytoplasmic/nucleoplasmic localization similar to that of *At*AMI1 (Figure 3), all of the examined enzymes are indeed functionally related with respect to their enzymatic activity. They all catalyze the conversion of IAM to IAA* in vitro*, albeit with different specific activities (Table 1). Nonetheless, our enzymatic studies provided clear evidence to support their recognition as plant PAM/IAM-amidohydrolases. Together with the observation that the *N. tabacum* homolog of *AtAMI1* also encodes for a functional IAM-hydrolase [27], the presented results highlight a widespread occurrence of IAM-hydrolases throughout the plant kingdom. This is supportive of a conserved and presumably basic role of AMI1-like IAM-hydrolases in plant development and a tentative contribution to auxin biosynthesis in particular.

### 2.4. Occurrence and Auxin Activity of Phenyl-2-acetic Acid in Arabidopsis

**Figure 6:**
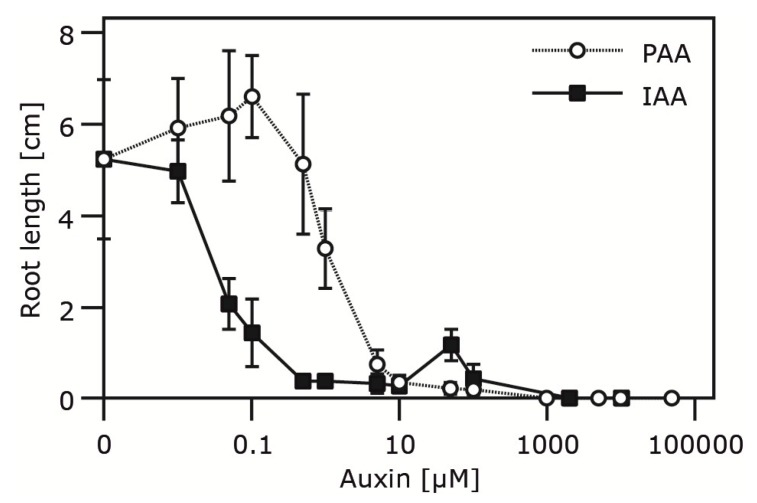
Impact of PAA and IAA on primary root elongation.

The determination of substrate specificities of the examined amidases revealed a particular preference for the conversion of PAM to PAA. To investigate this aspect in closer detail, we first tried to analyze whether PAA has an impact on the root development of sterilely grown *Arabidopsis* seedlings, as earlier described for *P. sativum* [47], *Avena sativa* [48], and *T. majus* [5]. As shown in Figure 6, PAA exerts an auxin-typical effect on primary root growth. The required dose to phenocopy the impact of IAA, however, was approximately 50-fold higher. This finding is in agreement with previous work of Koepfli and colleagues [47], assigning PAA 5% of IAA activity in pea.

Root elongation was quantified in two-week old *A. thaliana* seedlings grown on medium containing indicated concentrations of either IAA or PAA. Seedlings were removed from the petri dishes, and root length was recorded. At least ten seedlings for each condition were measured. Shown is the absolute root length in cm for either IAA or PAA treated plants. The error bars indicate the standard error of the mean (SE).

**Figure 7:**
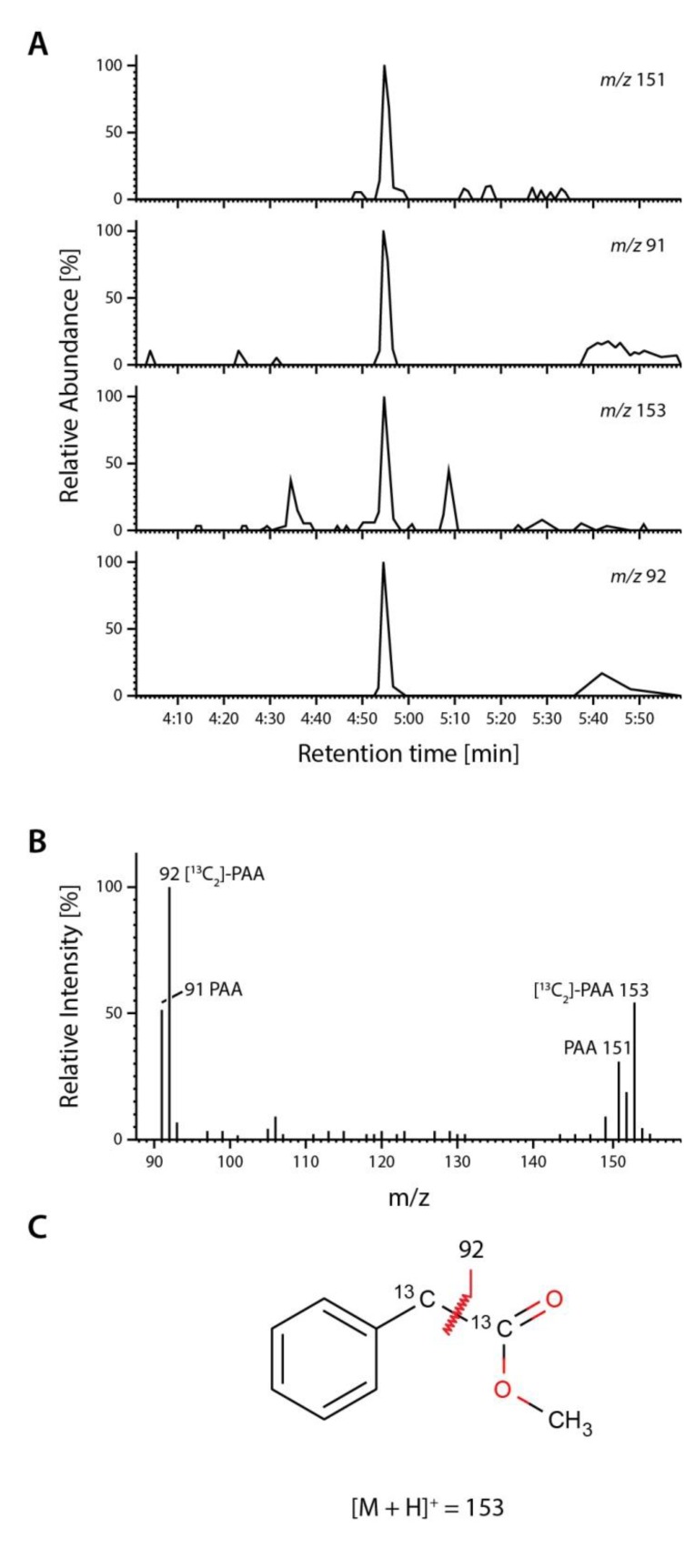
Detection of endogenous PAA. Endogenous PAA in sterile-grown *A. thaliana* was analyzed by extracting the organic compounds from rosettes of two-week old plants with boiling methanol in the presence of 1 nmol [^13^C_2_]-PAA standard (Sigma). (**A**) The extract was pre-purified by solid-phase extraction and analyzed by GC-MS. Under these conditions, PAA elutes at 4:55 min. The upper panel shows the extracted ion chromatograms for endogenous PAA (*m*/*z* 91 and 151) and stable isotope labeled [^13^C_2_]-PAA (*m*/*z* 92 and 153); (**B**) The middle panel shows the characteristic full-scan mass spectrum for co-chromatographed endogenous PAA (*m*/*z* = 91, 151) and [^13^C_2_]-PAA (*m*/*z* = 92, 153); (**C**) Fragmentation and structure of the [^13^C_2_]-PAA methyl ester. As the two ^13^C atoms in [^13^C_2_]-PAA are attached to the acetate side chain, which is cleaved during ionization, only the mass of the parent ion of the labelled standard shows a shift of +2 atomic mass units relative to the endogenous compound. In consequence of fragmentation, however, the mass of the fragment of the standard PAA is shifted by only +1 atomic mass unit.

Secondly, we determined the steady-state levels of PAA and IAA in leaf tissue of both sterilely grown and soil grown *Arabidopsis* plantlets. After purification and analysis of the methylated extracts, PAA was identified as an endogenous compound in *A. thaliana* by full-scan mass spectrometry (Figure 7).

Next, the content of PAA in two- and six-week old plants was investigated (Table 2). Previous reports give a free IAA content of approximately 30 to 60 pmol (g FW)^−1^ for two-week old *Arabidopsis* seedlings [21,49]. In contrast, the PAA content in leaf tissue of sterile grown plants was slightly higher in two-week old plantlets. This is in contrast to an observed 10- to 100-fold lower concentration of PAA relative to IAA in *T. majus* [5]. The presented results may hint at the dynamic nature of auxin concentrations that have been demonstrated within the plant and throughout its life cycle.

**Table 2:** PAA contents in two-week old and six-week old leaf tissue of *A.* *thaliana* ^a^.

Plant Age (Weeks)	Free PAA pmol (g FW)^−^^1^	Free IAA pmol (g FW)^−^^1^
Two weeks	98 ± 6.4	35 ± 2.7
Six weeks	23 ± 2	15 ± 1

^a^ Shown are means ± SD from three independent experiments.

## 3. Experimental Section

### 3.1. Plant Material and Plant Growth Conditions

All experiments were carried out using either *A. thaliana* (L.) Heynh. ecotype Col-0 (originally from Nottingham *Arabidopsis* Stock Centre, NASC, stock N1092), *O. sativa* L. cv. Millin (obtained from Yanco Rice Research Institute, Yanco, NSW, Australia), *S. bicolor* (L.) Moench *var.* Redland, *M. truncatula* Gaert. cv. Jemalong A17, or *P. trichocarpa* Hook. (Botanical Garden, Ruhr-University Bochum, Germany). While leaf material from poplar was directly taken from mature trees grown in their natural environment, *S. bicolor* and *M. truncatula* seeds were sown onto a mixture of soil and sand (2:1) and cultivated in a greenhouse at 22 to 24 °C during daytime and 18 to 20 °C over night, with a 16 h light/8 h dark cycle. Unless stated otherwise, rice was grown in Murashige and Skoog medium [50] containing 3% (w/v) sucrose and 0.3% (w/v) gelrite (Duchefa, Netherlands). The photosynthetically active radiation was no less than 150 μE·m^−2^·s^−1^ (supplementary light, if required, from sodium-vapor lamps).

### 3.2. RNA Isolation and RT-PCR

Total RNA was extracted with TRIzol reagent (Invitrogen), and the first strand cDNA was synthesized from 2 μg of total RNA using Oligo(dT)_15_ primer (Promega) and an AMV Reverse Transcriptase (Promega). The PCR parameters for the amplification of the amidase genes were 95 °C for 10 min followed by 35 cycles of 95 °C for 45 s, 58 °C for 45 s, and 72 °C for 90 s; for the amplification of the coding regions, a proofreading polymerase (Pfu, Fermetas, St. Leon-Rot, Germany) was used. The primers used for PCR are given in Table 3. Obtained fragments were either subcloned into the vector pGEM-T easy (Promega) or pBluescript SK(+) (Stratagene) and sequence integrity was subsequently verified by commercial sequencing (GATC, Konstanz, Germany; Sequencing Service, Ruhr-University Bochum, Dept. of Biochemistry I, Bochum, Germany).

**Table 3:** Primers used for cloning of the described amidases.

Primer Name	Sequence (5'–3')
*Os*AMI1-SacI-His2C-For	5'-TATGAGCTCTATGGCGATGGCGGGTGGAG-3'
*Os*AMI1-KpnI-His2C-Rev	5'-TATGGTACCGTGATTGGAGGACCAAGTTTTAG-3'
SbAMI1-SpeI-pUC-For	5'-TATACTAGTATGGCGATGGGCGGCGATTAC-3'
SbAMI1-SmaI-pUC-Rev	5'-TATCCCGGGGAGAGAGGAGTCTGGTGAGC-3'
*Mt*AMI1-XhoI-His2B-For	5'-TATCTCGAGATGGAAACAGCCTCAGACTATG-3'
*Mt*AMI1-XbaI-His2B-Rev	5'-TATTCTAGATATTTTTCAATGTTATCATAAATACTC-3'
*Pt*AMI1-XhoI-His2B-For	5'-TATCTCGAGATGGAACGAGACCCGGATTATG-3'
*Pt*AMI1-XbaI-His2B-Rev	5'-TATTCTAGATATTTTTCAGTGATCTCAACCTG-3'
*Mt*AMI1-BglII-pUC-For	5'-TATAGATCTATGGAAACAGCCTCAGACTATG-3'
*Mt*AMI1-SalI-pUC-Rev	5'-TATGTCGACCTATTTTTCAATGTTATCATAAATACTC-3'

Restriction sites are underlined.

### 3.3. Generation of Bacterial Expression Constructs

All DNA fragments were amplified of total cDNA of the different species using PCR with gene-specific primers (Table 3). In case of the rice amidase, the DNA fragment was inserted into the expression vector pTrcHis2C (Invitrogen) by using the *Sac*I/*Kpn*I sites. The DNA fragments of the amidases from *Medicago* and poplar were integrated into the *Xho*I/*Xba*I site of the vector pTrcHis2B (Invitrogen). The *S. bicolor* amidase cDNA fragment was inserted into the *XhoI*/*HindIII* site of pTrcHis2A (Invitrogen). In the four resulting constructs pTrcHis2C-*Os*AMI1, pTrcHis2B-*Mt*AMI1, pTrcHis2B-*Pt*AMI1, and pTrc2A-*Sb*AMI1 a translational fusion of the amidases with a myc/(His)_6_ double-tag was generated.

### 3.4. Preparation of GFP Amidase Fusion Constructs

Constructs suitable for the analysis of the intracellular localization of the deduced amidase GFP fusion proteins were obtained by cloning the amidase fragments into pSP-EGFP [31]. The amidase fragment from pTrcHis2C-*Os*AMI1 was digested with *Sac*I/*Kpn*I and integrated into the same sites of pSP-EGFP. The *Sac*I/*Sal*I fragment from pTrcHis2B-*Pt*AMI1 was also cloned into pSP-EGFP, resulting in a *Pt*AMI1:GFP fusion. As all C-terminal GFP fusions of the *Medicago* amidase lacked* in vivo* fluorescence, a N-terminal GFP-tag was fused to this amidase by using the vector pUC-GFPn [51]. To obtain a suitable DNA fragment, the coding sequence was amplified by PCR, adding restriction sites for *Bgl*II and *Sal*I, respectively (Table 3). After subcloning of the resulting DNA fragment into pBluescript SK(+) and sequencing, the *MtAMI1* *Spe*I/*Sal*I fragment was introduced into pUC-EGFPn. In case of the *S. bicolor* amidase gene, the coding sequence was amplified using primers that added *Spe*I/*Sma*I sites to the corresponding DNA fragment. After cloning into pBluescript SK(+) and sequence analysis the *Spe*I/*Sma*I fragment was introduced into the *Spe*I/*Sal*I site of pUC-EGFPn.

### 3.5. Heterologous Expression of Recombinant Amidases

To express hexahistidine tagged amidases 300 mL of 2xYT medium were freshly inoculated (1:10) with appropriate over night cultures and incubated at 37 °C under constant shaking. Protein expression was induced with 1 mM IPTG when an OD_600_ of either 0.6, in case of the *Mt*AMI1 construct, or 1.3, for the *Os*AMI1 and *Pt*AMI1 constructs, was reached. The cultures were transferred to either 4 °C (*Mt*AMI1) or 30 °C (*Os*AMI1, *Sb*AMI1, *Pt*AMI1) allowing protein expression to occur for at least 18 h. Thereafter, bacteria were harvested by centrifugation (4,000 *g*, 15 min, 4 °C) and pellets were resuspended in 1/10 of the culture volume of lysis buffer (50 mM sodium phosphate buffer pH 7.5, 300 mM NaCl, 10 mM imidazole). The samples were then snap frozen in liquid nitrogen and then kept at −80 °C until further use. Complete cell disruption was subsequently achieved by a combination of incubation with lysozyme (45 min, 4 °C) and ultrasound (120 s). The soluble protein fraction, obtained by centrifugation (35,000 *g*, 15 min, 4 °C), was sterile filtered (0.22 μm) and used for Ni^2+^-affinity purification. The chromatography was carried out in accordance to the manufacturer’s protocol (Qiagen).

### 3.6. Transient Expression in Plants and Confocal Laser Scanning Microscopy

Transient expression and subsequent microscopic analysis was carried out on the basis of Pollmann* et al.* [31]. In brief, leaves from two- to three-week old *Arabidopsis* plants, grown on ½ MS plates [50] under environmentally controlled conditions, were bombarded with 1 μm gold particles at 6.5 bar pressure. The particles were emitted from a particle inflow gun as described by Finer and coworkers [52]. After bombardment, the plants were kept for one day under constant conditions (8 h light at 24 °C, 16 h darkness at 20 °C, photosynthetically active radiation 105 μE·m^−2^·s^−1^ from standard white fluorescent tubes). The transformed leaves were then analyzed with a confocal laser scanning system (ZEISS LSM 510 Meta).

### 3.7. Assay for Amidase Activity

Amidase activity was estimated by measuring the ammonia released from the amide substrates during the reaction as previously described [30]. In short, the different substrates (10 mM) were incubated with 5 μg of purified protein in 50 mM potassium phosphate buffer at pH 7.5, at 30 °C in a total volume of 0.3 mL. For background control, heat-inactivated enzyme (20 min, 100 °C) and empty-vector control samples were used. After an incubation time of 4 to 6 h, aliquots of 100 μL were taken and the reaction was stopped by adding 100 μL each of 0.33 M sodium phenolate, 0.02 M sodium hypochlorite, and 0.01% (w/v) sodium pentacyanonitrosyl ferrate(III) (sodium nitroprusside). After incubating for 2 min at 100 °C, each sample was diluted with 600 μL of water, and the absorbance was read at 640 nm. Each experiment was calibrated using NH_4_Cl solutions of known concentrations.

### 3.8. Root Growth Assay

The root growth bioassay was carried out according to the work of Zimmerman and Hitchcock [53]. Plants were grown aseptically on ½ MS medium [50] containing 1% sucrose, solidified with 0.8% gelrite (Duchefa, The Netherlands) or on the same medium supplemented with either 10 nM to 10 mM indole-3-acetic acid (IAA; from 100 mM stock in methanol) or 10 nM to 10 mM phenyl-2-acetic acid (PAA; from 100 mM stock in methanol). Plates were wrapped in gas-permeable surgical tape (BSN medical GmbH, Hamburg, Germany) and grown vertically under constant environmental conditions (8 h light at 24 °C, 16 h darkness at 20 °C, photosynthetically active radiation 105 μE·m^−2^·s^−1^ from standard white fluorescent tubes) for two to three weeks. Subsequently, the root lengths of at least ten individual plants per treatment were determined.

### 3.9. Auxin Extraction and Purification

The extraction and purification of auxins and other organic acids was carried out according to a previously described method [54]. Approximately 0.1 g of two- and six-week old *A. thaliana* leaf material was added to 1 mL of pre-warmed (65 °C) methanol and the extraction conducted for another 60 min at room temperature under gentle shaking. Prior to the extraction process each sample was supplemented with 1 nmol of [^13^C_2_]-PAA and 50 pmol of [^2^H_2_]-IAA (internal standard). Cell-free supernatants were dried under vacuum and pre-purified for subsequent gas chromatography-mass spectrometry analysis. For this, the dried residues were dissolved in 30 μL methanol to which 200 μL diethyl ether was added, followed by ultrasonic treatment (Sonorex RK510S; Bandelin, Berlin, Germany). The particle-free sample was then applied to a custom-made microscale aminopropyl solid-phase extraction-cartridge [55]. The cartridge was washed with 250 μL of chloroform:2-propanol = 2:1 (v/v), and the PAA containing fraction subsequently eluted with 400 μL acidified diethyl ether (2% acetic acid (v/v)). The eluates were taken to dryness using a vacuum centrifuge, re-dissolved in 20 μL methanol, and afterwards treated with 100 μL ethereal diazomethane and transferred to autosampler vials (Chromacol 05-CTV(A) 116; Fisher Scientific, Waltham, MA, USA). Excess diazomethane and remaining solvent were removed under a gentle stream of nitrogen, and the methylated samples were then taken up in 15 μL of chloroform.

### 3.10. Quantification of Endogenous Phenyl-2-Acetic Acid and Indole-3-Acetic Acid from Arabidopsis thaliana

Analysis was performed by GC-MS on a Varian GC 3400 gas chromatograph coupled to a Finnigan MAT Magnum system. Compounds were separated on a VF5ms column (Varian), 30 m, 0.25 mm ID, 0.25 μm film. The temperature program was 50 °C for 1 min, followed by an increase of 30 °C·min^−1^ to 150 °C, and kept at this temperature for 4 min. The temperature was further increased to 250 °C with a velocity of 20 °C·min^−1^. Thereafter the temperature was kept at 250 °C for 5 min. An aliquot of 1 μL of each sample was injected into the GC-MS system. PAA eluted at 4:55 min under these conditions, while IAA eluted at 12:15 min. Full scan spectra were recorded for the peaks co-eluting with authentic methylated standards and the mass spectra compared to spectra obtained from authentic standards. For the determination of free PAA, the molecular and tropylium ions at *m*/*z* 151/153 and 91/92, respectively (ions deriving from endogenous and [^13^C_2_]-PAA) were monitored. For IAA, ions at *m*/*z* 189/191 and 130/132 (ions deriving from endogenous and [^2^H_2_]-IAA) were analyzed.

### 3.11. Gel Electrophoresis and Immunoblotting

Denaturing gel electrophoresis was performed according to Laemmli [56]. The discontinuous systems consisted of 4% stacking gels and 12.5% resolving gels. Protein blotting onto nitrocellulose was carried out electrophoretically overnight (4 °C, 60 mA) as described by Towbin and colleagues [57]. Immunodetection followed standard procedures [58] with goat anti-rabbit IgG-conjugated alkaline phosphatase as the secondary antibody and 4-nitrotetrazolium blue and 5-bromo-4-chloro-3-indolyl phosphate as substrates. As primary antibody an α-AMI1-antiserum, previously described [31], have been used.

### 3.12. Phylogenetic Analysis

The phylogenetic tree was inferred using the Neighbor-Joining method [59]. The percentage of replicate trees in which the associated taxa clustered together was calculated by a bootstrap test with 500 replicates [60]. The evolutionary distances were computed using the Poisson correction method [61] and are in the units of the number of amino acid substitutions per site. All positions containing gaps and missing data were eliminated from the dataset (Complete deletion option). Phylogenetic analyses were conducted in MEGA5 (Molecular Evolutionary Genetics Analysis, MEGA Software).

### 3.13. Statistic Analysis

The data were analyzed with one-way ANOVA followed by Tukey’s B *post hoc* test to allow for comparisons among all means. Statistical analyses were conducted using PRISM version 5.03 (GraphPad Software).

## 4. Conclusions

On the basis of sequence similarity, it was possible to identify more than 40 AMI1-like candidate amidases in publicly available plant genome databases (Figure 2). Four *At*AMI1-orthologous, namely *Os*AMI1 (*O. sativa*), *Sb*AMI1 (*S. bicolor*), *Mt*AMI1 (*M. truncatula*), and *Pt*AMI1 (*P. trichocarpa*), were selected and closely analyzed.

Taking an* in vitro* approach, it was possible to provide evidence for their capability of converting auxin precursors like IAM, but also PAM, to naturally occurring auxins (Figure 5, Table 1). Our results infer that an amidase-dependent auxin biosynthesis pathway is likely to be widely established in plants to produce auxins. Intriguingly, our experiments revealed a specificity of the compared enzymes for PAM (Figure 5), which is a biosynthetic precursor of PAA, described to be endogenous to *P. mungo* [42,43]. PAA has been demonstrated to be endogenous to *A. thaliana* (Figure 7, Table 2), while we were not able to quantify PAM in the same plant with the equipment available in this study. Our findings confirm previous studies, which demonstrated that PAA is an endogenous constituent of many plant species including *P. mungo* [45], *S. lycopersicum*, *N. tabacum*, *Z. mays* [4], and *T. majus* [5].

PAA has long been known to trigger auxin-like effects (e.g., the initiation and stimulation of adventitious root growth [62,63]), although with considerably lower activity relative to IAA [64,65]. Nevertheless, PAA may act as an effective auxin under some developmental circumstances, emphasized by the observation that it was more efficient in lateral root formation in pea seedlings than IAA [66]. In addition, PAA is assumed to have both antifungal and antibacterial properties [67,68,69]; it was isolated from culture extracts of *Azospirillium brasilense*, pinpointing an involvement in defense mechanisms, protecting this bacterial strain from other soil inhabitants like for instance *Agrobacteria* and thus providing an advantage for* A. brasilense* to survive in its natural habitat. A comparable protection mechanism for the defense against microbial pathogens could also be effective in plants.

It is generally accepted that the major portion of physiologically active auxin is produced via the indole-3-pyruvic acid-pathway [9,10]. On the basis of our results, however, we suggest that IAM/PAM-amidohydrolases may also contribute to auxin biosynthesis, most likely in a pathway parallel to the main route. Genetic work from Zhao and coworkers [70] highlights a possible role for an IAM hydrolase-dependent IAA biosynthesis, as they demonstrated that *yuc* double and triple mutants were rescued by the tissue specific co-expression of a bacterial tryptophan 2-monooxygenase (iaaM), but not by exogenous application of auxin. The authors claim that iaaM converts tryptophan to IAA once it is expressed in plants. This statement is, however, not entirely correct because several studies clearly showed that overexpression of iaaM (e.g., in petunia) produced very high levels of IAM in these mutants [71,72]. While IAM contents in control lines were determined to be <1 pg/g fresh weight, the IAM levels reached 2.8 up to 25 μg/g fresh weight in leaf tissue of transformed plants. In addition, it has been shown that the petunia plants transformed with the *p19S*::*iaaM* construct also contained around 11- to 12-fold higher IAA levels, which implicates the action of downstream acting indole-3-acetamide hydrolases, functionally homologous to the bacterial iaaH enzyme, capable of converting the accumulated IAM to IAA. Thus, effective conversion of IAM to IAA in the *yuc* mutants co-expressing the *iaaM* gene, but no additional *iaaH* gene, is implicated. Alternatively, it may be concluded that IAM by itself, or any other compound that is produced from IAM is responsible for rescuing the *yuc* phenotype. Most intriguingly, it has recently been shown that YUC6 is capable of converting not only IPyA to IAA, but to also accept phenyl pyruvate as substrate to produce PAA [73]. The results presented here may point towards a co-evolution of enzymes involved in auxin production to establish dual activity for the production of IAA and PAA, respectively. 

Currently, the elucidation of the* in vivo* function of *At*AMI1 is of utmost importance to our lab and preliminary data obtained from mutants overexpressing the protein support our conclusions. At this stage, however, we cannot yet postulate that the enzymatic activity of *At*AMI1 *in planta* is unambiguously linked to IAA production* in vivo*. Nevertheless, the results of the present study will help to further elucidate the enzymatic basis of auxin biosynthesis in plants.
